# Short rehabilitation training program may improve postural control in children with autism spectrum disorders: preliminary evidences

**DOI:** 10.1038/s41598-020-64922-4

**Published:** 2020-05-13

**Authors:** Simona Caldani, Paola Atzori, Hugo Peyre, Richard Delorme, Maria Pia Bucci

**Affiliations:** 1 UMR 1141 NeuroDiderot Inserm, Paris University, Robert Debré Hospital, Paris, France; 2 Pediatric Balance Evaluation center, ENT Department, APHP, Robert Debré Hospital, Paris, France; 3 Child and Adolescent Psychiatry Department, APHP, Robert Debré Hospital, Paris, France; 4 High Functioning Autism Expert Centre, Fundamental Foundation, Paris, France; 5 Human Genetics & Cognitive Functions, Institute Pasteur, Paris, France

**Keywords:** Neurological disorders, Psychiatric disorders

## Abstract

Autism Spectrum Disorders subjects (ASD) is characterized by postural control deficits. This study aimed to explore the effect of a short postural rehabilitation training program on postural capabilities in children with ASD. Two groups (G1 and G2) of twenty children with ASD of IQ-, sex- and age- matched (mean age 11.7 ± 2.4 years) were included in this study. Posture was recorded by using the Balance Quest from Framiral on unstable platform in three different viewing conditions. The rehabilitation program consisted in two distinct postural control training exercises. Postural recordings were performed twice at T1 and T2 for both groups of children. Between T1 and T2 a 6-minute postural training was performed by the G1 group only, while the G2 group had a 6-minute of rest. Children were allocated randomly to the G1 or G2 groups. At T1, postural instability was similar for both groups of ASD children (G1 and G2) desp+\ite viewing conditions. At T2, we observed an improvement of postural control related to a mixed effect of training rehabilitation but also of test-retest. Knowing the potential of new rehabilitation strategies, the impact of postural control deficit in ASD children needs to be reconsidered. Well design case-control studies are requested to ensure scientific validity of postural rehabilitation training program.

## Introduction

Autism Spectrum Disorders (ASD) are neurodevelopmental disorders characterized by social communication and social interaction deficits, associated with the presence of restricted, repetitive and stereotyped behaviors^1^. Sensorimotor deficits as well as fine and gross motor skill impairment are consistently reported in children with ASD and correlated with the severity of social communication impairment^2,3^. Sensory abnormalities are frequently the earliest identifiable clinical features of developmental trajectory impairments^4,5^. Similarly to social communication deficit, motor impairments may represent core features of autism when considering a broader spectrum of symptom^6^.

Several groups measured postural sway in children with ASD and showed that affected children were significantly more unstable than children with neurotypical development^7,8^.

A larger surface area of the center of pressure (COP) was observed in children with ASD with respect to control participants^9–13^, associated with an increased COP velocity^10,11,14^.

Postural instability in children with ASD was also more significant when somatosensory inputs were affected, such as standing on an unstable platform with foams under the feet or when wearing a vibrating apparatus on the neck^7,13,15–18^. All of these studies reported a significantly larger body sway displacement and/or faster sway velocity in subjects with ASD compared to peers with typical neurodevelopment. It was most likely due to a reduced ability of subjects with ASD to appropriately integrate somatosensory inputs into the cerebellum to deliver an accurate motor response allowing to control their body stability.

This hypothesis was in line with the direct measures of cerebellar impairment activity in ASD using functional neuroimaging^3^. The cerebellum plays a major role in controlling the postural stability^19^, as well as in achievement of gross and fine motor skills^17^. The learning of new motor abilities appeared to be dependent of an adaptive control loop^20^ involving the cerebellum *via* its ability to integrate complex levels of sensory signals^21^. The cerebellum also projects long-distance connections with the subcortical and cortical structures, and specifically with the striatum and pallidum^22^. With the cerebellum, basal ganglia are involved in the regulation of motor and postural control by interplaying altogether in a homeostatic equilibrium^23^.

Several studies explored the efficacy of visual-postural training program in children, specifically in those with a cerebral palsy^24,25^. These authors reported significant improvement of postural stability after specific training strategies that encouraged motor abilities. Studies exploring the effect of visual-postural training in children with ASD are, at our knowledge, scarce. Smoot-Reinert *et al*.^13^ in a small group of children with ASD (n = 5) reported that 10 min of a vestibular swing was able to reduce postural sway, although no statistical analysis was performed. A recent study from Travers *et al*.^26^ explored in twenty-nine children with ASD the effect on postural control of balance training by using a video game they developed to allow a visual biofeedback. The authors reported that their balance training video game impacted the postural sway but also the postural stability.

In this study, we aimed to investigate the effect of a short visual-postural training rehabilitation in children with ASD. We previously reported poor postural control in these children, specifically in the context of somatosensory input deficits^27,28^. We thus hypothesized that children with ASD could improve their baseline postural instability by engaging them in a short training rehabilitation program based on their visual abilities.

## Materials and Methods

### Clinical characteristics of children tested

Two groups (G1 and G2) of twenty children with ASD, age-, sex- and IQ-matched were included in the study (see Table 1). Children were included at the Robert Debré pediatric university hospital in Paris (France), and underwent to a neurological exam. None of them reported any personal history of sensory deficit (in any of the five senses) and they were naïve of any psychotropic drugs. The best estimated diagnosis of ASD was based on DSM-5 criteria requiring to gather clinical information from the Autism Diagnostic Interview-Revised (ADI-R) and the Autism Diagnostic Observation Schedule (ADOS) associated to the clinical expertise. Their cognitive abilities were also assessed using the Wechsler Intelligence Scale for children (WISC-IV). The allocation of a subject to a specific group (G1 or G2) was generated in an unpredictable random sequence and the sequence was implementing in a way that concealed the treatments (rest vs. rehabilitation) until patients have been formally assigned to one of the two groups.

**Table 1:** Clinical characteristics of the two groups (G1 and G2) children with autism spectrum disorder (ASD) enrolled in the study.

	G1 N = 20	G2 N = 20
Age (years), mean (SD)	11.7 (2.4)	11.8 (2.1)
Diagnosis of ASD		
Autism Diagnostic Interview-Revised scores		
*Social Reciprocal Interaction*, *mean (SD)*	18.5 (4.2)	17.2 (3.9)
*Communication*, *mean (SD)*	12.0 (4.1)	11.9 (3.5)
*Stereotyped Patterns of Behaviors*, *mean (SD)*	4.8 (2.5)	4.9 (2.8)
Autism Diagnostic Observation Schedule scores		
*Social Reciprocal Interaction*, *mean (SD)*	8.0 (3.4)	7.9 (2.8)
*Communication*, *mean (SD)*	4.1 (1.4)	4.2 (1.6)
Cognitive assessment		
WISC-IV sub-test scores		
Verbal Comprehension, mean (SD)	95.7 (27.8)	96.5 (25)
Perceptual Reasoning, mean (SD)	90.2 (23.1)	92.2 (22)
Working Memory, mean (SD)	88.5 (20.1)	90.1 (21)
Processing Speed, mean (SD)	87.2 (19.9)	86.3 (18.1)

The investigation followed the principles of the Declaration of Helsinki and was approved by our Institutional Human Experimentation Committee (Comité de Protection des Personnes CPP Île-de-France). Written consent was obtained from the children’s parents after the experimental procedure was explained to them.

### Postural control test

The Multitest Equilibre system (www.framiral.fr) was used to evaluate postural stability^29,30^. The displacement of the Center of Pressure (CoP) was sampled at 40 Hz and digitized with 16-bit precision. Postural recording was performed on unstable platform in which the platform was moved by oscillations allowing forward and backward translations, with a constant linear velocity which may vary from 0.03 m/s to 0.07 m/s with a frequency of 0.25 Hz.

In a dark room, child stood on the Framiral platform, positioned on the platform footprints, to fix a small red bright target at a distance of 2.5 m. Children were instructed to stay as still as possible with their arms along their body and to fix the target. We tested three visual conditions: eyes open fixating a target (EO), eyes closed (EC) and eyes open with perturbed vision by optokinetic stimulation (OPTO). During optokinetic stimulation children have to look forward without fixating a target, while the optokinetic field is rotating on all the wall of the room, giving a sensation of visual vection. This impaired visual input affects the balance leaving vestibular inputs only to acting to control postural sway. The duration of each postural recording was 30 s, with a 15 s rest period between conditions to reduce the possible effects of tiredness. The order of the conditions was randomized. These measures were realized two times, T1 and T2, respectively before and after 6 min of postural control rehabilitation training for the subjects allocated to group G1, and before and after 6 min of rest (*i*.*e*. without any training) for the group G2.

### Rehabilitation training protocol for postural control

**Figure 1:**
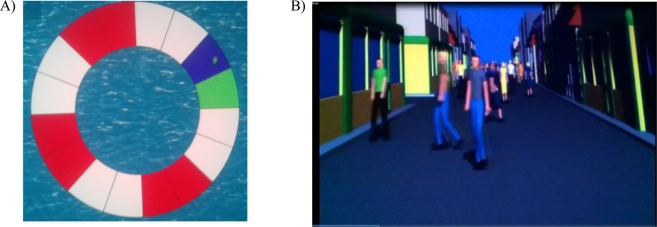
Postural rehabilitation training program: the buoy (**A**) and the crowd (**B**).

After the first postural recording (T1), children with ASD from G1 were trained for postural control rehabilitation by using two distinct postural control training exercises: the buoy and the crowd. Child stood on the Framiral platform and looked at a screen (340 cm × 170 cm) projected at a distance of 2.5 m. A buoy (130 cm × 130 cm) in the sea is projected on the screen (Fig. 1A). The body movements of child are expressed by a green point on the buoy. The goal is to move his/her body (that is the green point) on specific portions of the buoy avoiding to swim in the sea^31^. The second training type consisted on passers that were walking in a street towards to the child with a mean velocity ranging from 0.5 mm/s to 1.5 mm/s^29^ (Fig. 1B). Child had to move his/her body efficiently to avoid the passers-by.

The protocol of the two sessions of training was explained to the child and a test of 30 s was realized to verify that she/he had well understood the instructions. Then the training duration was of 3 minutes for each type of postural control training. Child stood on the Framiral stable platform and looked at a screen (340 cm × 170 cm) projected at a distance of 2.5 m. The two postural control training were run randomly.

After that, another postural recording was performed to estimate the main postural control parameters (T2). For children with ASD assigned to G2, they were not trained and the second postural measure (T2) was done after a six-minute of rest.

### Data analysis

Postural control performance was evaluated using the surface area of the CoP (in cm^2^), the mean velocity (mm/s) and the postural instability indices (PII). The surface of the CoP was calculated corresponding to the area of an ellipse encompassing 90% of all CoP data point excursions. A larger surface area of the CoP means a poor postural control. Mean velocity is an efficient indicator to quantify the neuro-muscular activity required to regulate postural control^32^: high values of mean velocity correspond to an important muscular effort to maintain the postural control. The PII is a global postural instability index used during routine test by clinicians. PII is = Σx Σy PI (F1, F2, F3)/CT (F1, F2, F3), where PI and CT are the spectral power index and cancellation time for each of the three frequency ranges (F1: from 0.05 to 0.5 Hz, F2: from 0.5 to 1.5 Hz, and F3: higher than 1.5 Hz). When PII is higher, the postural stability is weak^33^.

### Statistical analysis

ANOVA was run between the two groups (G1 and G2) of children with ASD on postural parameters (the surface area and mean velocity of the CoP and the PII) recorded two times (T1 and T2) under three distinct visual conditions (EO, EC and OPTO). Post-hoc comparisons were made with the Bonferroni’s method. Significance was considered when the p-value was below 0.05. All statistical analyses were processed using Statistica software (StatSoft, Inc.).

## Results

### The surface of CoP (cm²)

**Figure 2:**
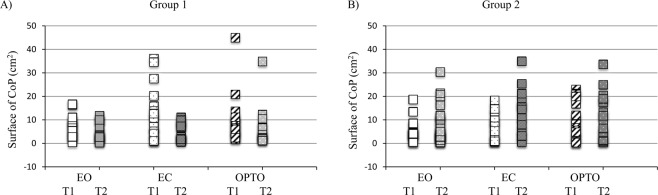
Individual values of surface area of centre of pressure (CoP) (cm^2^) in the three visual conditions tested (EO, EC and OPTO) during the two postural measures (T1 and T2) for both groups of children (**A**) for G1 and (**B**) for G2).

Figure 2 showed the *surface of CoP* covered in the three visual conditions (EO, EC and OPTO) at T1 and T2 for the two groups of children. The ANOVA showed a significant interaction T x G effect (F_(1,38)_ = 10.85, p < 0.002). Bonferroni post hoc test showed that the surface of CoP at T2 was higher for G2 than for G1 (p < 0.04) in the absence of any significant difference between G2 and G1 at baseline (T1). The ANOVA also reported a significant visual condition effect (F_(2,76)_ = 7.04, p < 0.001). Post hoc analysis revealed the surface of CoP was higher in EC with respect to EO conditions (p < 0.001), in accordance with recurrent findings in the literature showing the postural control was dependent from somatosensory inputs.

### Mean velocity of CoP (mm/sec)

**Figure 3:**
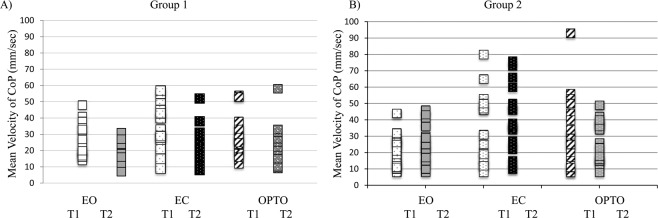
Individual values of the mean velocity of centre of pressure (CoP) (mm/sec) in the three visual conditions tested (EO, EC and OPTO) during the two postural measures (T1 and T2) for both groups of children (**A**) for G1 and (**B**) for G2).

Figure 3 showed the mean velocity of CoP (mm/sec) in the three visual conditions at T1 and T2 for the two groups of children. The ANOVA showed a significant interaction T x G effect (F_(1,38)_ = 16.88, p < 0.0002). Bonferroni post hoc test showed that the mean velocity of CoP for G1 was smaller at T2 than at T1 (p < 0.0001) suggesting an improvement of postural stability. This improvement was probably related to a mixed effect of training rehabilitation but also of test-retest since the ANOVA reported a significant time condition effect (F_(1,38)_ = 7.97, p < 0.007): the mean velocity of CoP was significantly smaller at T2 than those reported at T1 whatever the group considered (G1 or G2).

We also observed a significant visual condition effect (F_(2,76)_ = 10.68, p < 0.0001). The mean velocity of CoP in EO condition was significantly smaller than those reported in EC and OPTO conditions (p < 0.0001 and p < 0.03, respectively), which was also concordant with the positive effect of somatosensory integration on postural stability.

### Postural Instability Index (PII)

**Figure 4:**
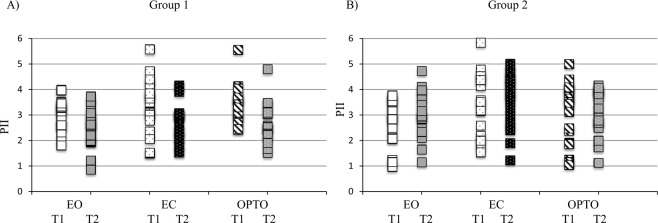
Individual values of the postural instability index (PII) in the three visual conditions tested (EO, EC and OPTO) during the two postural measures (T1 and T2) for both groups of children with (**A**) for G1 and (**B**) for G2).

The Postural Instability Index (PII) was also measured in the three visual conditions at T1 and T2 for the two groups of children tested (Fig. 4). We also reported a significant interaction T x G effect (F _(1,38)_ = 27.28, p < 0.0001) with a PII that was significantly higher at T1 than at T2 (p < 0.0001) in the G1 group. We however observed a similar effect for the G2 group but less meaningful since the PII was significantly smaller for G1 at T2 than for G2 at T2 (p < 0.05). The PII modifications were thus mediated by a mixed effect of training rehabilitation but also of test-retest.

The PII’s values were also dependent from the visual inputs (F_(2,76)_ = 12.07, p < 0.0001) with a reduced instability in the EO than in the EC and OPTO conditions (p < 0.0001 and p < 0.01, respectively).

## Discussion

Impairment in postural control is consistently reported in patients with ASD impacting the global functioning of subjects in their daily-life but also in their academic education. Our study aimed to explore the effect of short training rehabilitation program on postural control parameters. Interestingly, we observed an improvement of postural control in ASD related to a mixed effect of training rehabilitation but also of test-retest. We also reported that somatosensory inputs from visual origin improve the postural sway whatever the parameter of postural control considered.

The abnormalities of postural control in ASD we observed at baseline were concordant with previous findings in the literature^34^. We observed a global pattern of postural instability in subjects with ASD as suggested by a large surface area of the CoP compared to similar studies in children with neurotypical development. Also, we observed high mean velocities in subjects with ASD, which is correlated to the muscular effort requested to improve the postural control. These results were in accordance with previous findings by our groups^28^. Surprisingly, the rehabilitation program failed to improve the mean surface area of the CoP. In contrast, the group of children with ASD which did not undergo the visual-postural training reported a larger surface of the CoP at T2 compared to T1. Thus, this difference could be explained by fatigue, even if a break time of rest was included for each child. Additional studies with large sample are requested to allow the use of multivariate analysis taking account confounding variables.

Interestingly, we observed in our study that children with ASD benefited from a short visual-postural training program. In addition to the test-retest effect *i*.*e*. by the single effect of repetition, the body stability on the unstable platform was increased by additional rehabilitation strategies. Our results were in accordance with previous findings in the field of cerebral palsy^24,25^. Beyond the intensive repetition of a same motor sequence, specific rehabilitation training strategies seemed to increase significantly the postural stability as reported Pan *et al*.^35^ and Bahrami *et al*.^36^. In the field of autism or neurodevelopmental disorders, few studies have explored the effect of specific rehabilitation programs on postural control. One exploratory case report study^13^ observed a potential improvement of postural way after 10 min of a vestibular swing in a small group of children with ASD (n = 5). To our knowledge, only one case control study explored the effect of cognitive rehabilitation in ASD^26^. They reported that a video-based training program associated with a visual biofeedback may improve postural sway and stability in ASD.

Our results were very preliminary since limited by the size of our population, the design of the study (open case-control study, single set of pictures tested) and the delineation the rehabilitation protocol. We also did not include any children with neurotypical development as a control group. There was based on very preliminary evidence (data not shown) suggesting a ceiling effect of postural control performance in neurotypical children, *i*.*e*. their basal postural performances were very stable, even after rehabilitation strategies. We thus chose to consider in our study only children with ASD - not engage in the rehabilitation intervention program - as control participants. However, our results seemed in accordance with a recent brain imaging study with reported that short dynamic rehabilitation programs may impact the morphology of the brain in parallel to the improvement of the postural stability^37^. These latter reported in a group of young healthy adult volunteers, significant changes in the gray matter volume and in the white matter microstructure in several frontal and parietal regions, as well as in the right cerebellar white matter, after two 45 minute sessions of dynamic balance training. Specifically the cerebellar plasticity observed in this study may account for the rapid improvement of postural performance. Moreover, given the important implication of cerebellum in the cognitive processes we could suggest that our visual-postural training, that allows to stimulate children to use all sensory information and to better integrate them via the cerebellum, could have a beneficial impact not only on postural control but also on cognitive functions such as learning of language and working memory^38^. Further studies combining neuro-imaging studies with behavioral postural rehabilitation program may be useful in order to explore the substratum of such dynamic effect on postural control in children with ASD.

In this report we found that all children with ASD were more stable when they performed postural recording on unstable platform in the eye opened condition than in the two addition experimental conditions which impacted the somatosensory inputs (i.e. the eye closed or disturbed vision (with optokinetic stimulation)). Our findings were in line with the literature^39^ or from previous findings from our group^33^. Several studies showed that the presence of a visual disorders as well as strabismus^40,41^ or age-related macular degeneration^42^ could affect postural stability. These studies confirmed the importance of visual inputs for reaching body stability. Recall that postural control corresponds to a complex neurological function that relies on somatosensory inputs that are conveyed by the visual, proprioceptive, and vestibular system^39^. Our results were also congruent with the hypothesis of Carver *et al*.^43^. These authors suggested that adaptive mechanisms allow an optimal control of postural stability even when inputs from one somatosensory modality are defective by reweighting the integration processes from the others somatosensory input sources. In our study, patients were evaluated on an unstable platform, a postural condition in which proprioceptive inputs are altered. The improvement of postural control parameters in the eye-open condition we reported in our study may be explained by the reweight of the visual and vestibular information in the subjects we explored.

Postural control deficit which deeply impact subjects with ASD in their early acquisition needs to be reconsidered knowing the potential new rehabilitation strategies. Well design case-control studies are requested to ensure scientific validity of postural rehabilitation training program and delineate the optimal protocol.
